# Depletion of preexisting B‐cell lymphoma 2‐expressing senescent cells before vaccination impacts antigen‐specific antitumor immune responses in old mice

**DOI:** 10.1111/acel.14007

**Published:** 2023-11-23

**Authors:** Ozmen Cobanoglu, Lou Delval, Daniele Ferrari, Lucie Deruyter, Séverine Heumel, Isabelle Wolowczuk, Abir Hussein, Ayse Nur Menevse, David Bernard, Philip Beckhove, Frauke Alves, François Trottein

**Affiliations:** ^1^ CNRS, INSERM, CHU Lille, Institut Pasteur de Lille, U1019 ‐ UMR 9017 ‐ CIIL ‐ Center for Infection and Immunity of Lille University of Lille Lille France; ^2^ Translational Molecular Imaging Group, Max‐Planck Institute for Multidisciplinary Sciences Göttingen Germany; ^3^ Clinic of Hematology and Medical Oncology, Institute of Interventional and Diagnostic Radiology University Medical Center Göttingen Göttingen Germany; ^4^ Centre de Recherche en Cancérologie de Lyon, Inserm U1052, CNRS UMR 5286, Centre Léon Bérard, Université de Lyon Lyon France; ^5^ Department of Internal Medicine III University Hospital Regensburg Regensburg Germany

**Keywords:** aging, Bcl‐2, cellular senescence, immune responses, senolytics, tumor growth, vaccination

## Abstract

The age‐related decline in immunity reduces the effectiveness of vaccines in older adults. Immunosenescence is associated with chronic, low‐grade inflammation, and the accumulation of senescent cells. The latter express Bcl‐2 family members (providing resistance to cell death) and exhibit a pro‐inflammatory, senescence‐associated secretory phenotype (SASP). Preexisting senescent cells cause many aging‐related disorders and therapeutic means of eliminating these cells have recently gained attention. The potential consequences of senescent cell removal on vaccine efficacy in older individuals are still ignored. We used the Bcl‐2 family inhibitor ABT‐263 to investigate the effects of pre‐vaccination senolysis on immune responses in old mice. Two different ovalbumin (OVA)‐containing vaccines (containing a saponin‐based or a CpG oligodeoxynucleotide adjuvant) were tested. ABT‐263 depleted senescent cells (apoptosis) and ablated the basal and lipopolysaccharide‐induced production of SASP‐related factors in old mice. Depletion of senescent cells prior to vaccination (prime/boost) had little effect on OVA‐specific antibody and T‐cell responses (slightly reduced and augmented, respectively). We then used a preclinical melanoma model to test the antitumor potential of senolysis before vaccination (prime with the vaccine and OVA boost by tumor cells). Surprisingly, ABT‐263 treatment abrogated the vaccine's ability to protect against B16 melanoma growth in old animals, an effect associated with reduced antigen‐specific T‐cell responses. Some, but not all, of the effects were age‐specific, which suggests that preexisting senescent cells were partly involved. Hence, depletion of senescent cells modifies immune responses to vaccines in some settings and caution should be taken when incorporating senolytics into vaccine‐based cancer therapies.

## Graphical abstract

The potential impact of age‐related senescent cell removal on vaccine efficacy is elusive. Depletion of senescent cells prior to vaccination (prime/boost) had little effect on OVA‐specific antibody and T‐cell responses in old mice. In contrast, treatment abrogated the vaccine's ability to protect against B16‐OVA melanoma growth, an effect associated with reduced antigen‐specific T‐cell responses. Caution should be taken when incorporating senolytics into vaccine‐based cancer therapies.

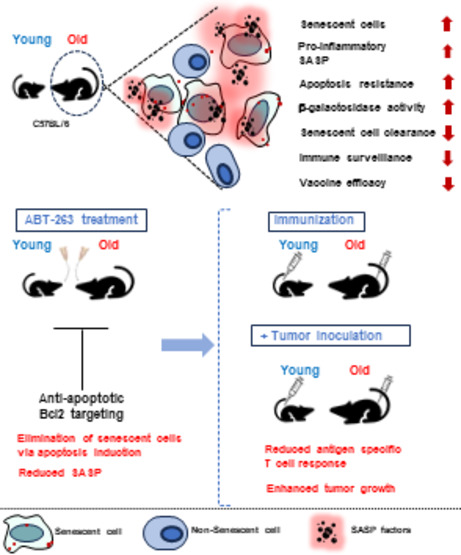

**Abbreviations**
Bcl‐2: B cell lymphoma‐2
DCs: dendritic cells
IL: interleukin
LNs: lymph nodes
LPS: lipopolysaccharide
MCP‐1: monocyte chemoattractant protein 1
NK: Natural killer
ODN: oligodeoxynucleotide
OVA: ovalbumin
SA‐β‐Gal: senescence‐associated β‐galactosidase activity
SASP: senescence‐associated secretory phenotype

## 1 INTRODUCTION

Vaccines are less effective in older people, due to a functional decline in innate and adaptive immune responses (Crooke et al., 2019; Nikolich‐Žugich, 2018). Changes in the numbers and functional activity of various immune cells (ranging from antigen‐presenting cells to effector cells) contribute to age‐related defects in immunity (Nikolich‐Žugich, 2014). The cellular and molecular impairments affecting the innate immune system include dysregulated signaling cascades and altered transcriptional programming, which notably reduce the host's ability to respond to adjuvants (e.g., pathogen‐associated molecular patterns). With regard to the adaptive immune system, the involution of primary lymphoid organs (thymus) and reduced cellularity in the bone marrow impair B and T lymphopoiesis and thus contribute to an age‐associated fall in the relative abundance of naive T cells (mostly CD8^+^) and B cells (Crooke et al., 2019; Nikolich‐Žugich, 2014; Nikolich‐Žugich, 2018). Aged B and T cells also display intrinsic changes in their activation threshold, effector capacity, homeostasis, and trafficking (Frasca et al., 2017; Lee, Fra‐Bido, et al., 2022; Nikolich‐Žugich, 2014). Along with age‐related changes in the microenvironment (lymphoid organs and non‐lymphoid tissues) and levels of circulating factors, these changes weaken cellular (effector) immune responses and humoral immune responses in older individuals. A decline in the immune response with chronological aging is associated with a state of chronic, low‐grade inflammation status known as inflammaging, which is characterized by elevated concentrations of pro‐inflammatory cytokines like interleukin (IL)‐6 and IL‐1β. The accumulation of senescent cells is an important contributor to inflammaging in older individuals (Baker et al., 2011; Cai et al., 2020; Xu et al., 2018).

Cellular senescence is a permanent state of cell cycle arrest that occurs in proliferating cells exposed to various types of damage or stress, including inflammatory and metabolic insults, DNA damage, and telomere shortening. Senescent cells overexpress antiapoptotic molecules (such as the B‐cell lymphoma‐2 (Bcl‐2) family of proteins) and are resistant to cell death (Zhu et al., 2016). Although senescent cells do not proliferate, they are transcriptionally and metabolically active and secrete a range of pro‐inflammatory and immunomodulatory cytokines, growth factors, and proteolytic components as part of the senescence‐associated secretory phenotype (SASP), with potent effects on the surrounding cells and tissues (Birch & Gil, 2020; Childs et al., 2017; Gorgoulis et al., 2019). Cellular senescence has various physiological roles throughout life. First, it has an essential role in tissue and organ formation during embryonic development. Throughout adulthood, cellular senescence exerts functions in wound healing and protects against oncogenic insults.

With chronological aging, senescent cells accumulate as a result of several factors including elevated levels of stress factors and defective immune surveillance (Ovadya et al., 2018). The progressive accrual of senescent cells is responsible, partially via the induction of a chronic SASP, for many aging‐related disorders: chronic inflammation, a decline in the regenerative potential and function of tissues, degenerative disorders, and cancer (Van Deursen, 2014). Experiments on mouse models have shown that the removal of senescent cells (using pharmacological and/or genetic approaches) prevents the onset of (or at least attenuates) age‐related disease pathologies and increases life span and fitness (Baker et al., 2016; Chang et al., 2016; Farr et al., 2017; Ogrodnik et al., 2021). Therefore, therapeutic strategies designed to target and deplete senescent cells (i.e., senolytics) have gained attention in the field of aging research and hold great promise for the treatment of age‐related dysfunction (Chaib et al., 2022; Kirkland & Tchkonia, 2020). Senolytic agents include natural, diet‐derived factors (such as certain flavonoids, albeit with nonspecific activity) and synthetic inhibitors of the antiapoptotic molecules that are upregulated in senescent cells. Senolytic drugs can be divided broadly into two main classes, depending on the mechanism of action; kinase inhibitors and Bcl‐2 family inhibitors (Kirkland & Tchkonia, 2020). As a result of the promising research in mouse models mentioned above, senolytics have been used in the clinic to treat senescence‐related diseases (Chaib et al., 2022; Di Micco et al., 2021). For safety reasons, the first senolytic treatment to be studied in humans was a combination of dasatinib (a multi‐tyrosine kinase inhibitor) and quercetin (a flavonol that targets the PI3K/AKT pathway) (Kirkland & Tchkonia, 2020). This cocktail was well tolerated and was shown to remove senescent cells, decrease local and systemic inflammation, and alleviate physical dysfunction in idiopathic pulmonary fibrosis (Hickson et al., 2019; Justice et al., 2019). These results prompted the initiation of large clinical trials of treatments targeting fundamental aging processes and senescence‐related diseases.

With regard to the clinical value of senolytic drugs, it is essential to investigate the latter's potential effects on vaccine efficacy. To our surprise, we were unable to find any publications on the consequences of senolytic treatment on vaccine efficacy in old mice. In view of the putative harmful effects of senescent cells on the immune system (Lorenzo et al., 2022; Palacio et al., 2019), one can speculate that senescent cell depletion might lead to an increase in vaccine efficacy. In contrast, one must bear in mind that certain senescent immune and/or stromal cells might have key roles in immune responses to vaccines. In the present study, we investigated the effects of senolysis (using the drug ABT‐263) on the humoral and cellular immune responses in vaccinated, old mice and (control) young mice. ABT‐263 targets the Bcl‐2 family of prosurvival proteins (Zhu et al., 2016) and is already used to treat cancer in the clinic. We sought to determine whether or not ABT‐263 would interfere with the immune response to the administration of two difference ovalbumin (OVA)‐containing vaccines. One vaccine contained Quil‐A® (a saponin‐based adjuvant known to induce antibodies in aged mice) and the other contained a CpG oligodeoxynucleotide (ODN) adjuvant. To study the effect of senolysis on the antitumor response, OVA‐vaccinated mice were grafted with OVA‐expressing B16 melanoma cells (antigenic boost by tumor cells). We found that ABT‐263 treatment depleted senescent cells and abrogated the production of systemic, SASP‐related factors in old mice. In the classical vaccine prime/boost system, and compared to vehicle‐treated old animals, ABT‐263 treatment had no significant effect on antigen‐specific antibody (slightly reduced) and T‐cell (slightly augmented) responses. In the vaccine prime and tumor boost model, ABT‐263 treatment was associated with low antibody production and T‐cell response, although these effects were not statistically significant. Strikingly, the depletion of senescent cells prior to vaccination was associated with a low vaccine's ability to restrict tumor growth. Some (but not all) of these effects were age specific. We conclude that the senolytic drug ABT‐263 influences the immune response and strongly affects the antitumor response in vaccinated, old mice.

## 2 RESULTS

### 2.1 ABT‐263 inhibits LPS‐induced, SASP‐related cytokine production by old splenocytes in vitro

**FIGURE 1:**
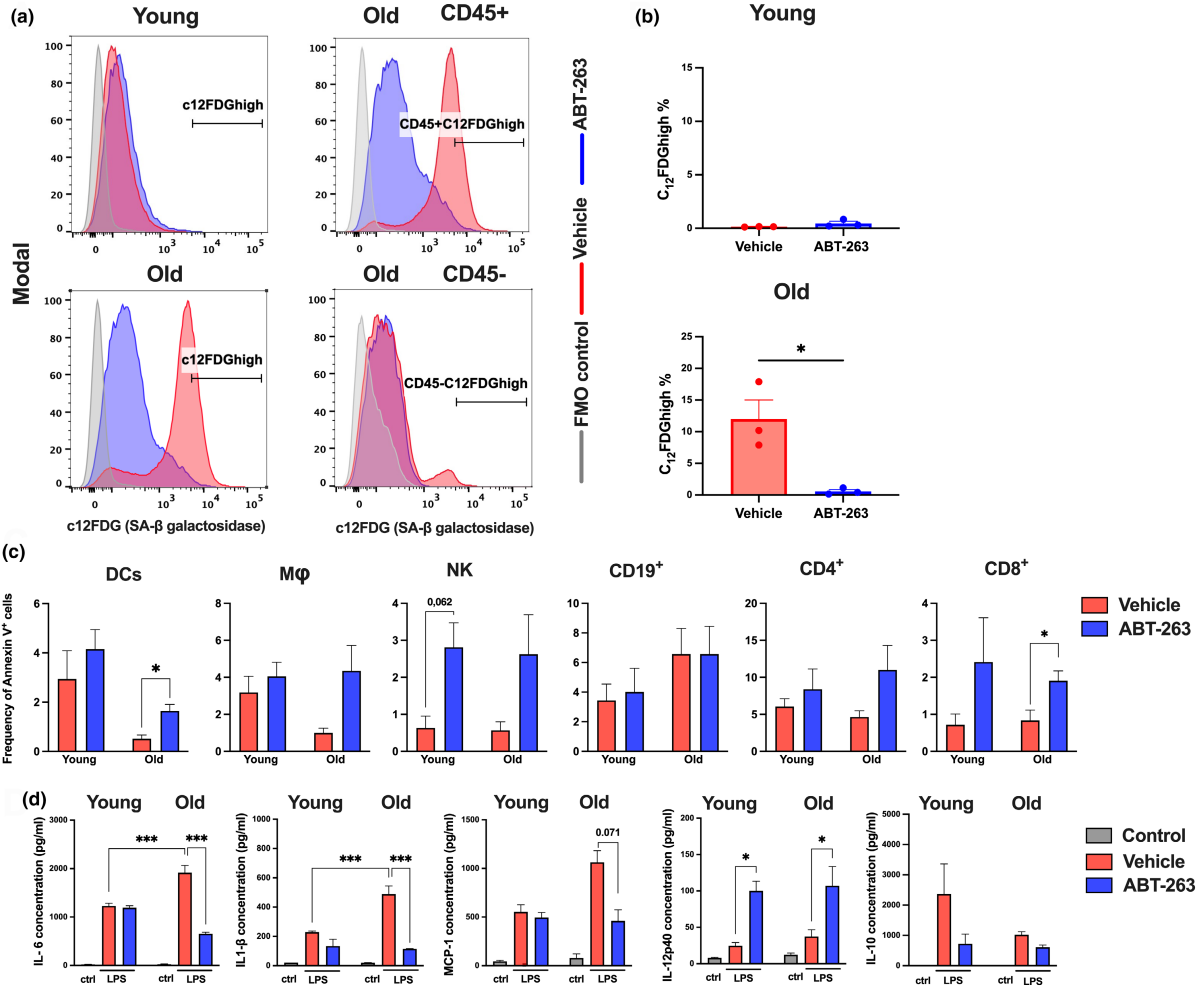
Depletion of senescent cells by ABT‐263 and consequences on LPS‐induced production of SASP‐related cytokines by old splenocytes. (a) Splenocytes collected from young and old mice were incubated with the fluorescent β galactosidase substrate CF_12_FDG and detection of the fluorescent substrate cleaved by β‐galactosidase was performed by flow cytometry. Representative profiles are depicted (*upper* panel: the CD45‐positive fraction and *lower* panel: the CD45‐negative fraction). (b) *Left* panel, Old splenocytes were cultured in the presence of ABT‐263 (1 μM) for 24 h and SA‐β‐galactosidase activity was assessed by flow cytometry. The percentages of labelled cells are indicated. (c) Frequencies of annexin V positive cells among each cell population (4 h post ABT‐263 treatment). (d) Splenocytes were treated with vehicle or ABT‐263 (1 μM) for 24 h and then stimulated with LPS at 1 μg/mL. After 24 h, supernatants were collected and cytokine production was determined by ELISA. For all graphs, errors indicate mean (*n* = 3–4). One representative experiment out of two (a–c) or three (d) are depicted. Significant differences were determined using the unpaired *t* test (b and c) and the two‐way ANOVA Tukey's multiple comparisons test (d) (*p < 0.05; **p < 0.01; ***p < 0.01).

We first assessed the effects of senescent cell depletion on the production of SASP‐related pro‐inflammatory cytokines by old murine immune cells ex vivo. To this end, cells were isolated from the spleen—a major secondary lymphoid organ involved in successful immune responses. Even though old (22‐month‐old) mice had a larger spleen, the number of splenocytes collected was lower than in young (2‐month‐old) mice (Figure S1a). Although young and old animals did not differ significantly in terms of the frequencies of splenic T cells and dendritic cells, old mice had lower proportions of B cells and natural killer (NK) cells and higher proportions of macrophages and neutrophils (Figure S1b). Lysosomal senescence‐associated β‐galactosidase activity (SA‐β‐Gal) is a good marker of cellular senescence (Cai et al., 2020). Using flow cytometry with the fluorogenic β‐galactosidase substrate C12FDG, we detected β‐galactosidase activity in splenocytes from old mice and in none of the splenocytes from young mice (Figure 1a,b; Figure S1c for the gating strategy). Most of the β‐galactosidase activity was detected in CD45‐positive (hematopoietic) cells.

ABT‐263, which is toxic at high doses in non‐senescent cells, was used to deplete senescent splenocytes. To avoid cytotoxicity as a confounding factor, we determined the ABT‐263 dose level at which >90% of the splenocytes collected from young adults were viable after 24 h of treatment. As a result, cells were treated with 1 μM ABT‐263 in subsequent experiments (Figure S1d). The proportion of old splenocytes expressing SA‐β‐Gal was strongly reduced after ABT‐263 treatment (Figure 1b, *lower* panel). The proportion of old dendritic cells (DCs), macrophages, NK cells and T cells (CD4^+^ and CD8^+^) positive for annexin V augmented after ABT‐263 treatment (Figure 1c). Some, but not all, of the effects were age‐specific.

Having established ABT‐263's ability to eliminate senescent cells ex vivo, we then stimulated ABT‐263‐treated splenocytes with lipopolysaccharide (LPS) ‐ a well‐known Toll‐like receptor 4 agonist. As expected, LPS induced the production of interleukin (IL)‐6, IL‐1β, monocyte chemoattractant protein 1 (MCP‐1), IL12p40, and IL‐10 (Figure 1d). Upon LPS stimulation, the production of the prototypical SASP‐related factors IL‐6, IL‐1β, and MCP‐1 was higher in splenocytes from old animals than in splenocytes from young animals. Regarding immune regulatory cytokines, the LPS‐induced production of IL‐12p40 was identical in young and old splenocytes, while that of IL‐10 was lower in old splenocytes. ABT‐263 treatment prior to LPS stimulation dramatically restricted the secretion of IL‐6, IL‐1β, and MCP‐1 by old splenocytes but not by young splenocytes. Intriguingly, ABT‐263 treatment was associated with greater IL‐12 production by young and old splenocytes but lower production of IL‐10 (a cytokine with pleiotropic immunosuppressive effects) by the young splenocytes. These results show that the ex vivo depletion of ABT‐263‐sensitive cells among old splenocytes is associated with lower production of LPS‐induced, SASP‐related cytokines. The results also suggest that ABT‐263 has indirect effects, independently of the direct depletion of senescent cells.

### 2.2 ABT‐263 inhibits senescence‐associated signatures in old mice in vivo

**FIGURE 2:**
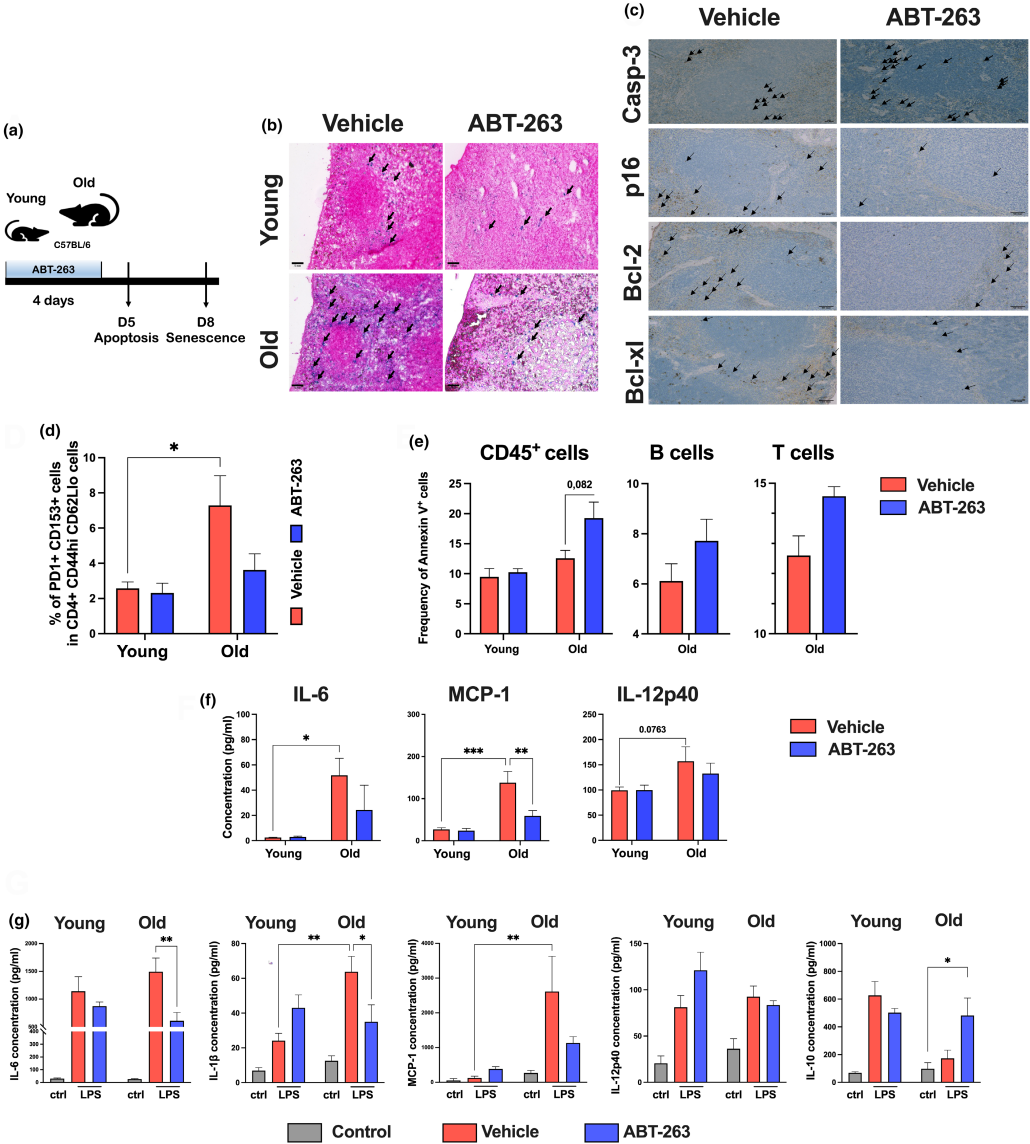
Effect of ABT‐263 treatment in the SASP signature in old mice. (a) Schematic procedure. Young and old mice were orally treated with ABT‐263 for 4 days. One day (Day 5) and 4 days later (Day 8), cell apoptosis and cellular senescence were respectively analyzed. (b) Representative images of SA‐β‐Gal staining of spleen sections. Arrows depict positive cells. (c) Representative images of cleaved caspase‐3 (Day 5) and p16, Bcl‐2, and Bcl‐xl (Day 8) staining of spleen sections. Arrows depict positive cells. (d) The percentages of PD1^+^ and CD153^+^ cells within CD4^+^ CD44^high^ CD62L^low^ cells are depicted for the four animal groups (*n* = 6–12) (Day 8). (e) Frequencies of annexin V positive cells among CD45+ cells (*n* = 3) (Day 5). (f) Cytokines in blood were quantified by ELISA (*n* = 3–5) (Day 8). (g) Splenocytes (Day 8) were stimulated with LPS (1 μg/mL). Supernatants were collected and cytokine production was determined by ELISA (*n* = 7). Pooled results from two independent experiments (d and g) and one of two representative experiments (b, c, e and f) are shown. Significant differences were determined using two‐way ANOVA Tukey's multiple comparisons test (*p < 0.05; **p < 0.01; ***p < 0.01).

We next analyzed the effect of ABT‐263 on senescence signatures in vivo. To this end, old animals were gavaged for 4 days with 50 mg/kg ABT‐263 (a standard protocol in the mouse system (Chang et al., 2016) and euthanized on Day 5 and 8 (Figure 2a). Beta‐galactosidase staining of spleen sections from old mice revealed a higher number of SA‐β‐Gal‐positive cells, relative to young counterparts (Figure 2b, *left* panel). It is noteworthy that most SA‐β‐Gal‐positive cells were located in the marginal zone around the lymphoid follicles, a region with high frequencies of B cells and macrophages. ABT‐263 treatment led to a reduced proportion of SA‐β‐Gal‐positive cells in old spleen (Figure 2b, *right* panel). This associated with enhanced expression of cleaved caspase‐3 (a marker of apoptosis) as assessed by immunohistochemistry (Figure 2c, *upper* panel). The presence or absence of the prototypical senescence marker p16^INK4a^ (referred to hereafter as p16) was then assessed using immunohistochemistry. p16‐positive cells accumulated in old spleens and were located at the same sites as SA‐β‐Gal‐positive cells (Figure 2c, *middle left* panel). Few p16‐positive cells were detected in young spleens (Figure S2a). Old mice treated with ABT‐263 had fewer p16‐positive cells than controls did (Figure 2c, *middle right* panel). The immunohistochemistry experiments indicated that levels of Bcl‐2 and Bcl‐xL expression were far higher intense in the spleen of old mice than in the spleen of young counterparts (Figure S2b). ABT‐263 treatment resulted in lower intensities of Bcl‐2 and Bcl‐xL labelling in aged spleen (Figure 2c, *lower* panels). To substantiate ABT‐263's impact in vivo, we analyzed the drug's effect on senescent CD4^+^ T cells (defined as CD44^high^ CD62L^low^ PD1^+^ CD153^+^ in the mouse system (Yoshida et al., 2020). As depicted in Figure 2d and Figure S2c (for the gating strategy), senescent CD153^+^ CD4^+^ T cells were detected in old spleen but not in young spleen. ABT‐263 treatment reduced the frequency of CD153^+^ CD4^+^ T cells, albeit not significantly (*p* = 0.20). Analysis of annexin V expression revealed a higher (almost twofold, *p* = 0.082) level of apoptosis in CD45‐positive cells collected from ABT‐263‐treated old mice relative to controls (Figure. 2e). B cells and T cells were particularly affected (not significant) (Figure. 2e; Figure S2d and not shown). It is noteworthy that 4 days posttreatment, ABT‐263 did not overtly influence the proportion of splenocytes in old mice, although a slight reduction of macrophages was noted (Figure S2e).

Aging is associated with elevated systemic levels of inflammatory cytokines, and senescent cells are a major contributor to the inflammaging process (Cai et al., 2020). As expected, serum IL‐6 and MCP‐1 levels (IL‐1β was not detected) were much higher in old mice than in young mice (Figure 2f). ABT‐263 treatment strongly reduced the blood IL‐6 and MCP‐1 levels. In contrast, ABT‐263 treatment did not have a significant effect on IL‐12p40 expression (IL‐10 was not detected). Spleens from ABT‐263‐treated mice were collected, and the splenocytes were stimulated with LPS. The levels of IL‐1β and MCP‐1 production were higher in splenocytes from old animals than in splenocytes from young animals (Figure 2g). The same was true (albeit to a lesser extent) for IL‐6 and IL‐12p40. The anti‐inflammatory cytokine IL‐10 was less induced in old splenocytes. Remarkably, in vivo ABT‐263 treatment significantly reduced the production of IL‐1β, IL‐6, and (albeit to a lower extent) MCP‐1 in old mice. In contrast, in vivo ABT‐263 treatment had no effect on IL‐12p40 production by old splenocytes and was associated with greater IL‐10 production. Taken as a whole, these data showed that the in vivo removal of ABT‐263‐sensitive cells in old mice was associated with the impaired production of SASP‐associated cytokines.

### 2.3 ABT‐263 treatment prior to vaccination has a small impact on humoral and cellular immune responses in old mice

**FIGURE 3:**
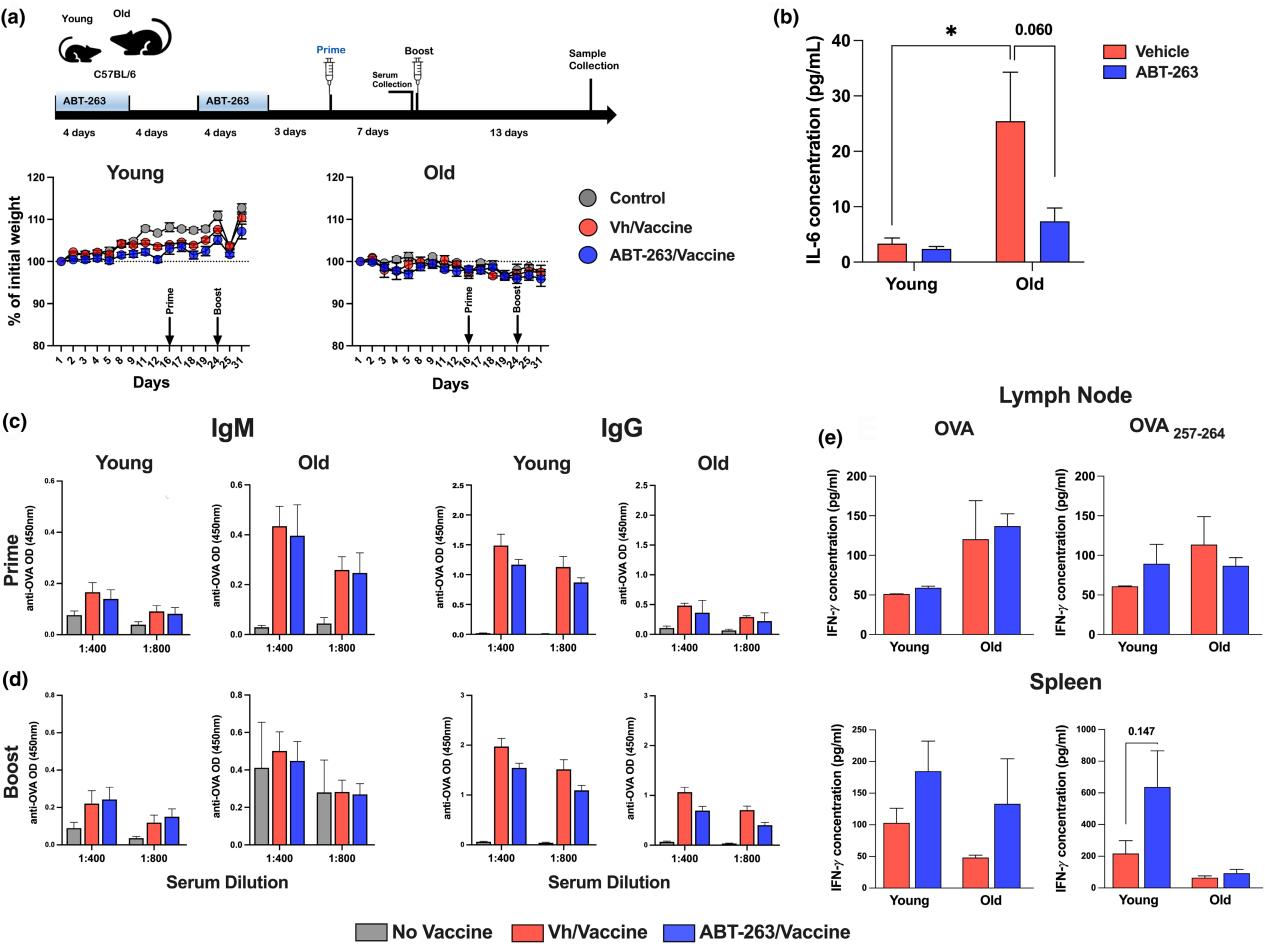
Effects of ABT‐263 treatment on humoral and cellular OVA‐specific immune responses in old and young mice. (a) *Upper* panel, schematic procedure (prime‐boost setting). Mice were orally treated with ABT‐263 for 4 days. After a 4‐day interval, the procedure was repeated. Three days after the last ABT‐263 inoculation, mice were immunized with the vaccine (prime). Animals were boosted 7 days later and sacrificed 13 days after the boost. *Lower* panel, Body weight was measured over the course of treatment and vaccine procedure (*n* = 5). (b) IL‐6 concentration in blood was quantified by ELISA (*n* = 5). (c and d) IgM and IgG titers were determined by indirect ELISA. Serum samples were collected after the prime (c) and after the boost (d) (*n* = 5). (e) LN cells and spleen cells from vaccinated mice were restimulated with whole OVA or OVA_257–264,_ for 48 h. IFN‐γ production was assessed by ELISA (*n* = 5). (a–e) One representative experiment out of two performed are depicted. Significant differences were determined using the two‐way ANOVA Tukey's multiple comparisons test (*p < 0.05).

To investigate the effect of senolysis on the immune response elicited by vaccination, old mice were treated twice with ABT‐263 during 4 days with a 4‐days interval between the two treatments (Figure 3a, *upper* panel). Mice were inoculated with the OVA Quil‐A® vaccine 3 days after the second ABT‐263 treatment and, a week later, received a booster vaccination. Samples were collected 13 days after the booster. To study the primary humoral response, we also collected serum after priming. ABT‐263 treatment did not significantly influence body weight gain in young animals and body weight loss in old animals (Figure 3a, *lower* panel). At sacrifice, the serum levels of IL‐6 and MCP‐1 in old mice were lower after ABT‐263 treatment—indicating that senolysis had a long‐term effect on chronic low‐grade inflammation (Figure 3b and Figure S3a). Accordingly, mRNA expression of the genes coding for p16 and Bcl2 was lower in ABT‐263‐treated, old mice than in controls (Figure S3b).

The antibody production induced by vaccination is a key parameter in vaccine effectiveness. Primary humoral immune responses and responses evoked by memory B cells are both altered in older individuals. We first analyzed the effect of senolysis on the primary humoral response. The anti‐OVA IgM response was much higher in old mice than in young animals, while the opposite was seen for anti‐OVA IgG (Figure 3c). This finding confirmed the presence of a class‐switching defect in old animals. Depletion of senescent cells prior to vaccination did not affect anti‐OVA IgM and IgG production in old and young mice. The secondary antibody response was then evaluated after the boost (Figure 3d). The anti‐OVA IgM response was similar in old mice and young animals, whereas the anti‐OVA IgG response was still less intense in old mice. In both age groups, ABT‐263 treatment did not significantly (slight reduction) modify the levels of OVA‐specific IgM or IgG. IgG1 (a T helper 2‐related isotype) was the dominant isotype in this context (Figure S3c). To examine the OVA‐specific T cell response, draining lymph node (LN) cells and splenocytes were restimulated with whole OVA or OVA peptides. LN cells (old but not young) and splenocytes (young but not old) collected from vaccinated animals produced interferon gamma (IFN‐γ) in response to whole OVA and/or to the predominantly MHC class I‐restricted peptide OVA_257–264_ (Figure S3d). Of note, IFN‐γ was not (LN) or hardly (spleen) detected in response to the MHC class II‐restricted peptide OVA_223–239_ (not shown). ABT‐263 treatment had no significant effect on IFN‐γ production by restimulated LN cells and spleen (slight augmentation) cells (Figure 3e and not shown). Hence, ABT‐263 treatment prior to vaccination (prime/boost) has no significant effect on humoral and cellular immune responses in old mice.

### 2.4 ABT‐263 treatment prior to vaccination accelerated tumor growth in aged mice

**FIGURE 4:**
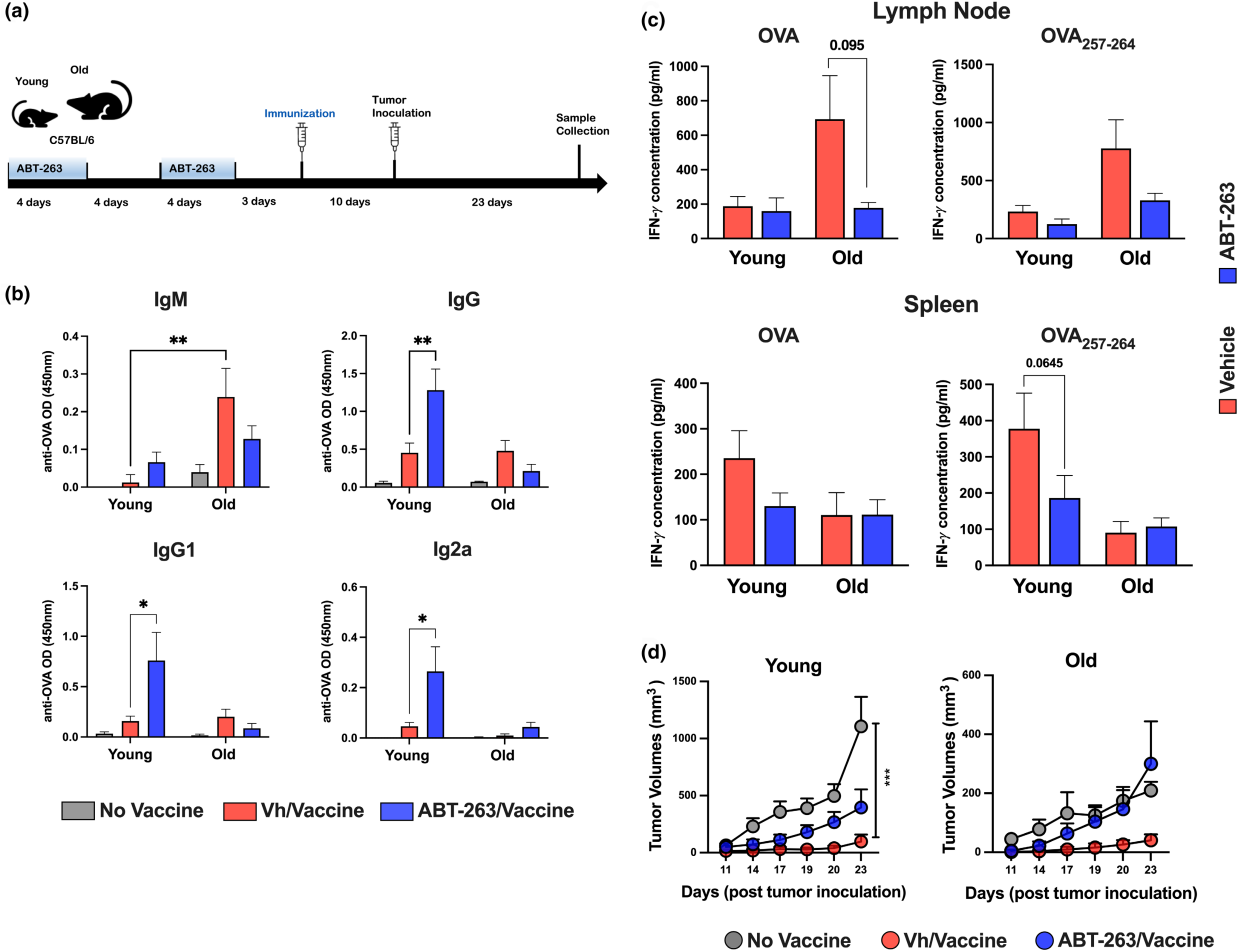
Effects of ABT‐263 treatment on OVA‐specific immune responses and B16‐OVA growth in young and old mice. (a) Schematic procedure (prime‐only regimen and challenge with B16‐OVA). Mice were orally treated with ABT‐263 for 4 days. After a 4‐day interval, the procedure was repeated. Three days after the last ABT‐263 inoculation, mice were immunized with the vaccine. Tumor cells were inoculated 10 days later and sacrificed 23 days after the inoculation. (b) IgM, IgG, IgG1, and IgG2a titers were determined by indirect ELISA (dilution: 1:800) (*n* = 3 for unvaccinated mice and *n* = 5 for vaccinated mice). (c) LN cells and spleen cells from vaccinated young and old mice were restimulated with OVA or OVA_257–264_ for 48 h. Supernatants were collected and IFN‐γ were quantified by ELISA (*n* = 10). (d) B16‐OVA tumor volumes were measured over time (*n* = 6 for unvaccinated mice and *n* = 10 for vaccinated mice). (a and b) A representative experiment out of two is depicted. (c and d) A pool of two representative experiments is depicted. Significant differences were determined using the two‐way ANOVA Tukey's multiple comparisons test (*p < 0.05; **p < 0.01; ***p < 0.01).

To analyze the potential effects of senolysis on the antitumor response resulting from vaccination, CpG ODN was used as an adjuvant. Briefly, senescent cells were depleted (as shown in Figure 3) and mice were primed with OVA + CpG ODN 3 days after the discontinuation of ABT‐263 treatment (Figure 4a). Ten days after this priming, OVA‐expressing B16 tumor cells were engrafted. This vaccination regimen (comprising priming only and then a challenge with B16‐OVA) was effective in abrogating tumor growth in young animals (Figure S4a). Of note, ABT‐263 treatment had no effect on tumor growth in unvaccinated animals. The effect of ABT‐263 was then studied in young and old vaccinated mice. At the time of sacrifice (Day 23 after tumor inoculation), mRNA expression of the genes coding for p16 and Bcl2 was lower in ABT‐263‐treated, old mice than in controls (Figure S4b). The OVA‐specific IgM response was higher in old animals than in young animals (Figure 4b). In contrast, there was no difference in the IgG response (mostly IgG1) between the two age groups. Although ABT‐263 treatment prior to vaccination had no effect on the IgM response, it significantly improved the IgG response (both IgG1 and IgG2a) response in young animals. Strikingly, ABT‐263 treatment in old animals resulted in a lower (though not significant) IgM and IgG responses (Figure 4b; Figure S4c). The T‐cell response was then examined. Compared with LN cells from vaccinated young mice, LN cells from vaccinated old mice produced more IFN‐γ in response to both OVA and OVA_257–264_ (Figure 4c; Figure S4d). When the spleen cell response was examined, the opposite was observed. In response to OVA and OVA_257–264_, ABT‐263 treatment decreased (albeit not significantly) IFN‐γ production by old LN cells (Figure 4c). It also reduced (albeit not significantly) IFN‐γ production by young splenocytes whereas it had no effect on old splenocytes. Lastly, we investigated the potential effect of ABT‐263 treatment on subcutaneous B16‐OVA tumor growth. Relative to young animals, B16 melanoma tumor growth was delayed in old mice (Figure 4d). In this setting (a single immunization 10 days before the B16 graft), vaccinated old mice were partially protected against subcutaneous tumor outgrowth. Surprisingly, ABT‐263 treatment of old mice completely abrogated the vaccine's protective effect. The observation was slightly different in young mice; after ABT‐263 treatment, vaccinated young mice were still protected but to a lesser extent. We conclude that ABT‐263 treatment before vaccination influenced the immune response in old mice (with an impaired humoral and cellular response) and dramatically increased tumor outgrowth. Differences (in the humoral response) and similarities (in the T‐cell response and tumor development) were also observed in young animals—suggesting that the observed effects were underpinned by age‐dependent mechanisms and age‐independent mechanisms.

## 3 DISCUSSION

The age‐related decline in immune responsiveness is a critical issue for vaccine‐based prophylaxis and therapy in older adults. This decline is caused by combinations of impairments in various pathways, including cellular senescence (Agrawal & Weinberger, 2022; Budamagunta et al., 2021; Lee, Flores, et al., 2022; Palacio et al., 2019; Van Deursen, 2014; Yousefzadeh et al., 2021). Cellular senescence might be of particular interest in the setting of vaccination and is potentially amenable to prophylactic and/or therapeutic developments. To the best of our knowledge, the impact of senescent cell depletion on the vaccine response in older people or in preclinical models of aging has not been studied previously. Here, with the hope of alleviating immune dysfunction in older individuals, we used a senolytic approach. We focused on the broad Bcl‐2 family inhibitor ABT‐263, a drug currently used in the clinic to target apoptosis‐resistant tumor cells. Our objective was to explore this potential new senotherapeutic strategy for enhancing vaccine responses in older adults.

We initiated this study without any starting hypotheses, as the depletion of preexisting senescent cells might have both positive and negative effects on vaccine efficacy. As mentioned above, the aging process involves changes in (among others) immune cell numbers and functions, cellular organization in lymphoid organs and non‐lymphoid tissues, and levels of circulating factors that interact with both immune cells and their microenvironment to ensure appropriate immune responses (Frasca et al., 2017; Mittelbrunn & Kroemer, 2021; Nikolich‐Žugich, 2014). Age‐associated inflammation, which is due in part to a chronic SASP, blunts immunity in aged individuals (Freund et al., 2010; Goldberg et al., 2020; Pereira et al., 2020) Hence, the SASP might be a key element in the weak immune responses to vaccines. By eliminating preexisting senescent cells and thus by reducing levels of the SASP‐related inflammatory factors involved in inflammaging, one could expect to ameliorate vaccine responses. By way of an example, SASP factors reduce T‐cell proliferation and the B‐cell class switching and hypermutation required for the generation of high‐affinity antibodies (Frasca et al., 2014; Palacio et al., 2019). Decreasing the expression of immunosuppressive cytokines by senescent cells (IL‐10, for instance (Acosta et al., 2013)) might also improve immune responses. Moreover, the depletion of preexisting senescent cells in older individuals might enable danger‐associated signals in the microenvironment to act as adjuvants and promote effective immune responses. Hence, the use of ABT‐263 to eliminate age‐related senescent cells might conceivably have a beneficial effect on immune responses to vaccines. However, one must also bear in mind that in older individuals, almost all immune cells express senescent markers (including the bona fide p16)—albeit at different levels (Grosse et al., 2020; Hall et al., 2016; Martínez‐Zamudio et al., 2021; Yousefzadeh et al., 2021). These cells include macrophages, NK cells, T cells, and (memory) B cells. One should also consider that non‐senescent immune cells including T and B cells express Bcl‐2 members and that Bcl‐2 expression changes in aging (Delbridge et al., 2016; Rohner et al., 2020). Hence, ABT‐263 could eliminate critical (non‐senescent and/or senescent) immune cells and have a catastrophic effect on immune responses to vaccines. Our experimental data, however, do not support this hypothesis because ABT‐263 treatment did not abrogate immune responses, at least in the classical prime/boost protocol. One could also hypothesize that the removal of preexisting senescent cells could reduce the production of components required for immune responses after vaccination. During an immune response, senescence and energy‐sensing signaling pathways converge to regulate functional responses in immune cells—including B cells (Akbar, 2017). Some SASP factors signal to different immune cells. For instance, IL‐1α, IL‐6, IL‐7, and TGF are central players in CD4 T‐cell homeostasis, stimulation, and differentiation and thus have an impact on adaptive immune responses (Ben‐Sasson et al., 2009). The production of extracellular matrix proteins, proteases and growth factors (typically SASP factors) might also be important in immune responses triggered by the vaccine. The production of these factors by preexisting immune and nonimmune senescent cells might be important for vaccine‐associated immune responses.

To study the effect of senolysis on immune responses following vaccination, we designed an experimental protocol in which the effect of ABT‐263 was restricted to preexisting senescent cells and not new, stress‐induced Bcl‐2‐expressing cells. Indeed, studies of transgenic mice and gene‐targeted mice have revealed that Bcl‐2 family members have roles in immune responses (Smith et al., 2000). As ABT‐263 has a short in vivo half‐life (8–12 h) and the treatment regimen ended 3 days before vaccine administration in our experiments, it is unlikely that the treatment impacted the future immune response triggered by stressed Bcl‐2‐expressing cells (e.g., cells stressed by the adjuvant in the vaccine formulation). ABT‐263 treatment effectively removed senescent cells in old mice, as assessed by enhanced apoptosis, lower p16 and Bcl‐2 expression, a lower level of β‐Gal activity, and lower blood levels of SASP‐related factors. Despite the age environment, senescent cells (or at least those involved in the production of circulating SASP factors (Figure. 3b) failed to repopulate mice 23 days after the discontinuation of ABT‐263 treatment, which indicates that the drug had a long‐term effect. Immune responses to vaccines comprise several steps, including antigen presentation/processing by DCs (the sentinels of the immune system), DC maturation (a process that allows T‐cell activation after interaction with DCs), T‐cell expansion, T‐cell cytokine release, and antibody class switching. The removal of age‐associated senescent cells could have positive and/or negatively effects on certain obligate steps in the immune response. We developed two different adjuvant‐based protocols in order to investigate this question. Whatever the protocol used, we observed a slight decrease in antibody production by old mice after ABT‐263 treatment. Our findings contrast with literature reports in which the reduction of intrinsic levels of inflammatory cytokines (such as TNF‐α) led to an improvement in B‐cell function and an increase in antibody production (Frasca et al., 2014). Interestingly, a recent study showed that the treatment of old mice with the senolytic fisetin was associated with greater antibody production in response to a viral infection (Camell et al., 2021). It is noteworthy that after the administration of a vaccine containing CpG ODN, antibody production was enhanced—rather than reduced—in young ABT‐263‐treated mice (OVA expressed by B16 cells for the boost). This marked, age‐specific difference is difficult to interpret. Differences in exposure to OVA (due to differences in tumor growth) between young and old animals might have a role in the response to the boost. Depending on the vaccination protocol, the effect of ABT‐263 on the T‐cell response in old mice was different. In the classical OVA prime/boost regimen, no significant effects were observed. In contrast, when tumor cells served as booster of the immune response, ABT‐263 treatment reduced, albeit not significantly, the T‐cell response in old mice. As similar data were obtained with whole OVA and the MHC class‐I restricted OVA peptide, this suggests that CD8^+^ T cells were affected. These data are in line with a recent report showing reduced CD8^+^ T cell response in aged mice upon senescent cell depletion (dasatinib/quercetin cocktail) (Torrance et al., 2023). The global effect of ABT‐263 on the T‐cell response was not age‐dependent, as the results were similar in young and old mice (albeit differences occurred between LNs and spleen (Figure. 4c). In line with the reduced (CD8^+^) T‐cell response, tumor development was increased in ABT263‐treated vaccinated mice in general and in older mice in particular. Although senolytics have been shown to increase tumor growth in some settings (Kovacovicova et al., 2018), this effect is unlikely to occur in our setting because tumor cells were inoculated 13 days after discontinuation of the ABT‐263 treatment. We are now seeking to determine whether this effect is due to enhanced immunosuppression.

Rejuvenation of the immune system in older individuals is an attractive approach for improving vaccine effectiveness; various targets have been suggested, including mammalian target of rapamycin (Mannick et al., 2018). Our data show that ABT‐263 treatment before vaccine inoculation (classical vaccine prime/boost system) has little effect of antigen‐specific B‐cell and T‐cell responses. In contrast, in the vaccine prime and tumor boost model, ABT‐263 treatment impairs the humoral and cellular immune responses and worsens tumor development outcomes in vaccinated, old mice. Some of these effects were more marked in old mice (e.g., tumor growth) or only observed in old mice (e.g., less antibody production) which indicates that the removal of preexisting senescent cells had a causal role. However, it is clear from our data that ABT‐263 also acts independently of age‐related senescent cells. This is particularly true for the enhanced B‐cell response, which was observed only in young animals. The mechanisms underpinning this enhanced response have not been identified and warrant future study. Small numbers of senescent cells are found in young individuals (Jeon et al., 2022). In our experiments, the effects of ABT‐263 in young mice might be due to the removal of these senescent cells. The drug might also have effects on non‐senescent cells. It remains to be established, both in young and aged mice, how ABT‐263 affects immune cells, the senescent cell populations within immune cell subgroups, as well as the senescent cell populations in the parenchymal tissues relevant to immune functions and tumor growth.

Although our results provide novel insights into the effect of senolytics on vaccine efficacy, the present study had some limitations. Indeed, mouse data should be interpreted with caution and might not necessarily be extrapolatable to the situation in humans. Another limitation of the present study relates to our focus on a restricted set of adjuvants and on ABT‐263, a drug that mainly targets Bcl2 family members. The specific targeting of age‐related resident senescent cells should now be studied by applying other Bcl‐2 inhibitors (such as ABT‐199 and ABT‐737) or other classes of senolytics that do not target Bcl‐2. The senotherapeutic potential of fisetin or the dasatinib/quercetin cocktail—treatments that have different mechanisms—should be investigated. Along with pharmacological strategies, the selective depletion of p16‐expressing or p21‐expressing cells in transgenic mouse models might be instructive. Studies with senomorphics, which modulate senescent cell functions and interfere with the SASP to prevent inflammaging, might also provide additional information. Lastly, in the current study, we designed a short‐term senolytic treatment with continuous (daily) inoculation of the drug in aged mice. It would be interesting to design a prolonged treatment (e.g., once or twice monthly) in middle‐aged mice (e.g., Farr et al., 2017) and investigate the effects of chronic drug administration in vaccinated aged mice. In conclusion, and bearing in mind the abovementioned limitations, our data indicate that the senolytic drug ABT‐263 cannot—at least in the mouse model—be considered for use as an adjuvant for enhancing antitumor immune responses upon vaccination in the aged individual. In the context of cancer vaccines, ABT‐263 should be used with caution. Further preclinical and clinical research is needed to determine the age‐related influence of senescent cells on vaccine efficacy.

## 4 EXPERIMENTAL PROCEDURES

### 4.1 Mice and ethics statement

Specific pathogen‐free C57BL/6J mice (2‐month‐old and 22‐month‐old, male) were purchased from Janvier (Le Genest‐St‐Isle). All experiments complied with the current national and institutional regulations and ethical guidelines (Institut Pasteur de Lille/B59‐350009). Protocols were approved by the regional Animal Experimentation Ethics Committee (CEEA 75), and the French Ministry of Higher Education and Research (authorization numbers: 00357.03 and APAFIS#23718‐2020012112087749 v4).

### 4.2 In vivo ABT‐263 treatment

ABT‐263 was purchased from Clinisciences (Nanterre, France) and dissolved in DMSO for preparation of stock solutions and kept at −20°C. For in vivo treatments 10% ABT‐263 was formulated in 30% polyethylene glycol 400 and 60% Phosal 50 PG. Mice were treated by oral gavage (100 μL) with 50 mg/kg ABT‐263 or vehicle control administered for 4 consecutive days in two treatments 4 days apart. Cytokines were quantified by ELISA (Invitrogen and Biolegend).

### 4.3 Stimulation of mouse splenocytes

Red blood cells from cell suspensions were removed with Red Blood Cell lysis buffer. After washing, cell pellets were resuspended in RPMI medium supplemented with 10% fecal bovine serum (FBS), 1% penicillin/streptomycin. Splenocytes from aged and young mice (1 × 10^6^ cells/well) were treated in triplicates with ABT‐263 (1 μM, 0.1% DMSO) for 24 h in RPMI medium, washed and then stimulated with LPS (1 μg/mL). Cell viability was measured with XTT assay by following manufactures instructions (Cell Proliferation Kit II (XTT) Roche, MERCK). The colored formazan product was photometrically measured at 450 and 570 nm in a multiwell plate reader (Multiscan FC Microplate Reader). Analysis of annexin V expression was performed 4 h post‐ABT‐263 treatment.

### 4.4 Flow cytometry

Spleens were disaggregated into single cell suspensions by using a plunger from a sterile syringe to mince and stained for flow cytometric analysis. Samples were blocked with anti‐CD16/32 (Biolegend) for 30 min at 4°C and the following anti‐mouse primary antibodies incubated for 1 h at 4°C. All antibodies were from Biolegend: CD3:Pacific Blue, CD4:FITC, CD44:AF700, CD62L.PerCP/Cy5.5, PD1:PE, CD153:APC, PE isotype Ctrl, PerCP/Cy5.5 isotype Ctrl, APC isotype Ctrl (Figure. 2d). Single‐stained samples were prepared as compensations. Cells were washed twice and resuspended in PBS/2% FCS for analysis on LSR Fortessa using FlowJo v10.8 software (BD Life Sciences). Apoptosis induction by ABT‐263 was analyzed by flow cytometer using Annexin V‐FITC/PI staining kit (Biolegend).

### 4.5 Detection of SA‐β‐galactosidase activity

Cellular senescence was quantified using CF12FDG [5‐Dodecanoylaminofluorescein Di‐b‐D‐Galactopyranoside]. Briefly, lysosomal alkalinization of splenocytes was induced by preincubation of Bafilomycin A1 (100 nM) at 37°C for 1 h. 33 μM C_12_FDG was added in the culture media containing Baf A1 for another 2 h. The cells were washed three times in cold PBS before staining for FACS analysis. Frozen sections of spleen (5 μm‐thick) were used. Tissues were mounted in OCT embedding compound (Sigma Aldrich) and rapidly frozen in liquid nitrogen. Frozen sections were mounted on Superfrost adhesion microscope slides and washed twice in PBS. They were immersed in staining solution overnight at 37°C. Sections were rinsed, counterstained with eosin, rinsed, dehydrated, and cleared.

### 4.6 Immunohistochemistry

Tissue sections were stained with rabbit polyclonal anti‐cleaved Caspase‐3 (Cell Signaling), rabbit monoclonal anti‐p16, rabbit polyclonal anti‐Bcl‐2, or rabbit monoclonal anti‐Bcl‐XL (all from Abcam) antibodies. The slides were blocked for endogenous peroxidase with 3% H2O2 and boiled for antigen retrieval in citrate buffer (pH 6). Sections were incubated with the appropriate secondary antibody from Vector Laboratories (goat anti‐rabbit IgG antibody (H + L), washed, and incubated with the VECTASTAIN®Elite ABC Peroxidase standard kit (Vector laboratories, Newark, NJ). After several washes, the chromogen 3,3′‐diaminobenzidine from the Peroxidase Substrate Kit (Vector Laboratories) was added to each slide. The slides were counterstained with Mayer's Hemalun (Merck). Lastly, the slides were mounted with glycerin mounting medium (Dako). Images were acquired using an Evos M5000 microscope.

### 4.7 Gene expression analysis by RT‐qPCR

Quantitative RT‐PCR was performed exactly as described in (Delval et al., 2023). The murine primers used were as follows: *p16* (5′‐GCTCTGGCTTTCGTGAACATGT‐3′, 5′‐TTGAGCAGAAGAGCTGCTACGT‐3′ and (*bcl2*) (5′‐TCATGTGTGTGGAGAGCGTCA‐3′, 5′‐GATCCAGGTGTGCAGATGCC‐3′) and (*Gapdh*) (5′‐GCAAAGTGGAGATTGTTGCC‐3′, 5′‐ GCCTTGACTGTGCCGTTGA‐3′). Data were normalized against expression of the *Gapdh*.

### 4.8 Immunization and serum preparation

C57BL/6J mice were immunized subcutaneously on the right flank with 50 μg of endotoxin‐free OVA (Endofit, InvivoGen, Toulouse, France) plus 10 μg Quil‐A® (InvivoGen) or plus 20 μg CpG ODN 1826 VacciGrade (InvivoGen). The same procedure was repeated for the boost but with 25 μg OVA and 5 μg Quil‐A® or 10 μg CPG ODN per animal. Control mice were immunized with PBS. Blood samples were collected at the prime (facial vein) and at the day of sacrifice under anesthesia from external jugular vein. Samples were further separated by centrifugation at 10000 g for 10 min and serums were stored at −20°C until testing.

### 4.9 OVA‐specific T‐cell response and antibody detection by ELISA

Spleen and LN cells (1 × 10^6^ cells and 3 × 10^5^ cells/well, respectively) were restimulated with OVA (100 μg/mL) or the H‐2Kb restricted OVA‐derived peptide SIINFEKL (OVA_257–264,_ 2 μg/mL) for 48 h. IFN‐γ in supernatants was quantified by ELISA (Thermoscientific). OVA‐coated plates (96‐well high‐binding plates) were incubated with 50 μL of samples diluted in PBS containing 0.5% (w/v) BSA at 37°C for 2 h. After washes, anti‐mouse IgM and IgG HRP conjugate (ThermoFisher and Biolegend, 1:2000 (v/v) in diluent solution) were incubated for 1 h at 37°C. The color reaction was developed by adding TMB solution and the enzymatic reaction was stopped by adding 100 μL of 2 N H_2_SO_4_. Optical density was determined at 450 nm.

### 4.10 In vivo tumor growth

Fifty percent confluent B16‐OVA tumor cells were harvested using trypsin and subsequently prepared for injection by resuspending the cells in HBSS after washing. Mice were injected subcutaneously in the flank with 5 × 10^5^ tumor cells in 100 μL HBSS. Animals were monitored for body weights once daily starting at the day of the ABT‐263 treatment and continuing until the day of euthanasia. Tumor growth was monitored over time using microcallipers. Tumor volume (mm^3^) was calculated using the formula: 1/2 length (mm) × width (mm)^2^.

### 4.11 Statistical analysis

Results are expressed as the mean ± SEM (standard error of the mean) unless otherwise stated. All statistical analysis was performed using GraphPad Prism v9.2.0 software. Unpaired *t* test was used to compare two groups unless otherwise stated. Comparisons of more than two groups with each other were analyzed with the two‐way ANOVA Tukey's multiple comparison test. *p < 0.05; **p < 0.01; ***p < 0.001.

## AUTHOR CONTRIBUTIONS

FT conceived and supervised the study. OC designed the experiments, and performed the animal experiments. OC and DF analyzed the humoral response, LD performed immunohistochemistry, SH performed the RT‐PCR, AH and ANM performed the cell cultures. OC, LD, IW, DB, PB, FA, and FT analyzed the resulting data. OC and FT designed the figures and drafted the manuscript. All the authors revised the manuscript and provided critical comments. FT, LJC, PB, FA, and FT obtained funding.

## FUNDING INFORMATION

This project has received funding from the European Union's Horizon 2020 research and innovation program under the Marie Sklodowska Curie grant agreement No 861190 (PAVE). This project also partially benefited support from the French National Research Agency (Agence Nationale de la Recherche, ANR): AAP générique 2019, ANR‐19‐CE15‐0033‐01, DREAM (FT). This work was also supported by the Institut National de la Santé et de la Recherche Médicale, the Centre national de la Recherche Scientifique, the University of Lille, and the Pasteur Institute of Lille. OC received a PhD fellowship from the European Union.

## CONFLICT OF INTEREST STATEMENT

The authors declare that the research was conducted in the absence of any commercial or financial relationships that could be construed as a potential conflict of interest.

## Supporting information


Figure S1.

Figure S2.

Figure S3.

Figure S4.

## Data Availability

The data that support the findings of this study are available from the corresponding author upon reasonable request.
