# Supplementary figures and images for: Depletion of preexisting B‐cell lymphoma 2‐expressing senescent cells before vaccination impacts antigen‐specific antitumor immune responses in old mice
Source: Aging Cell. 2023 Nov 23;22(12):e14007. doi: 10.1111/acel.14007 (PMC10726819; doi:10.1111/acel.14007)

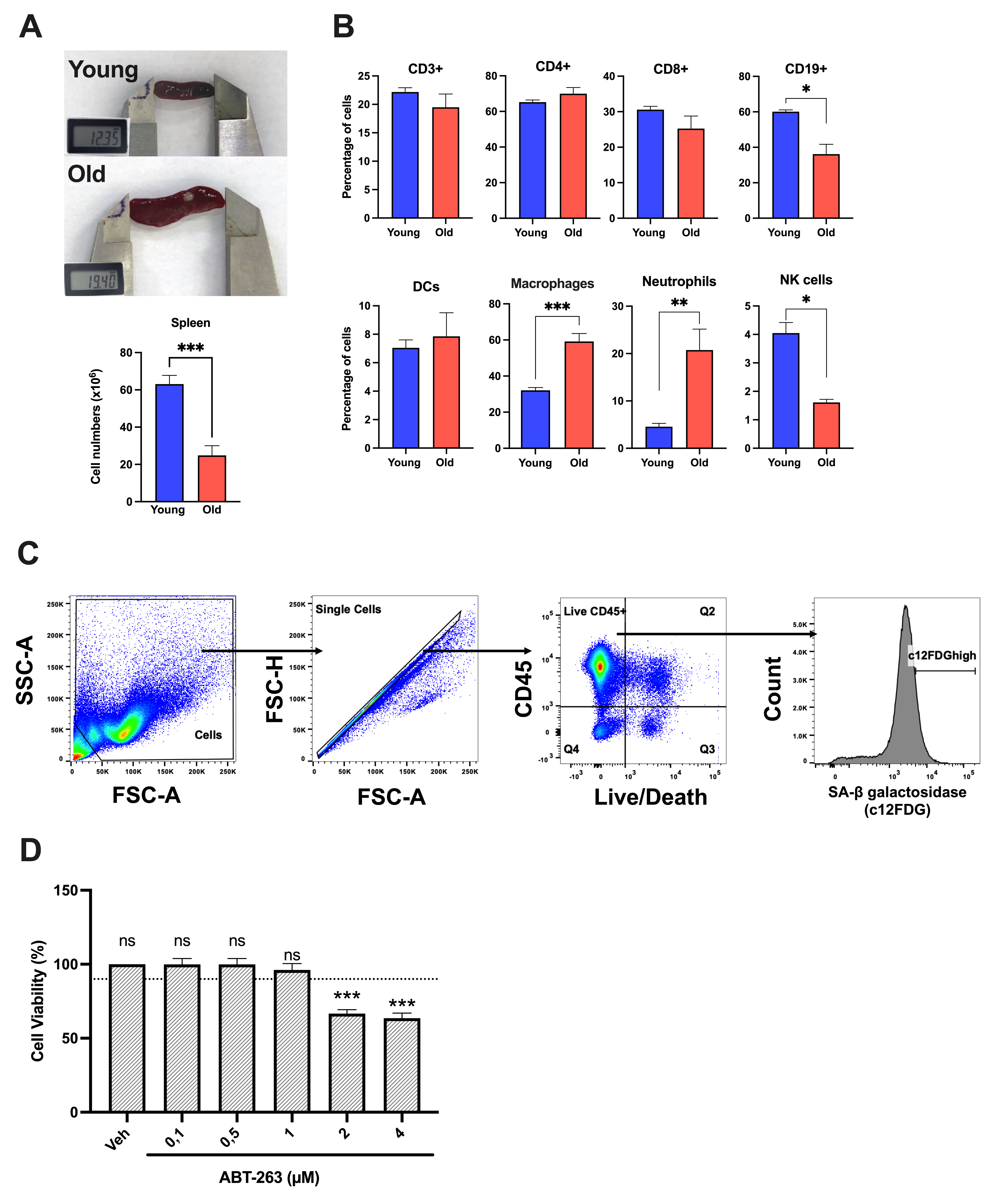

Supplement: Supplementary file 1 — Figure S1. [file ACEL-22-e14007-s003.tiff]

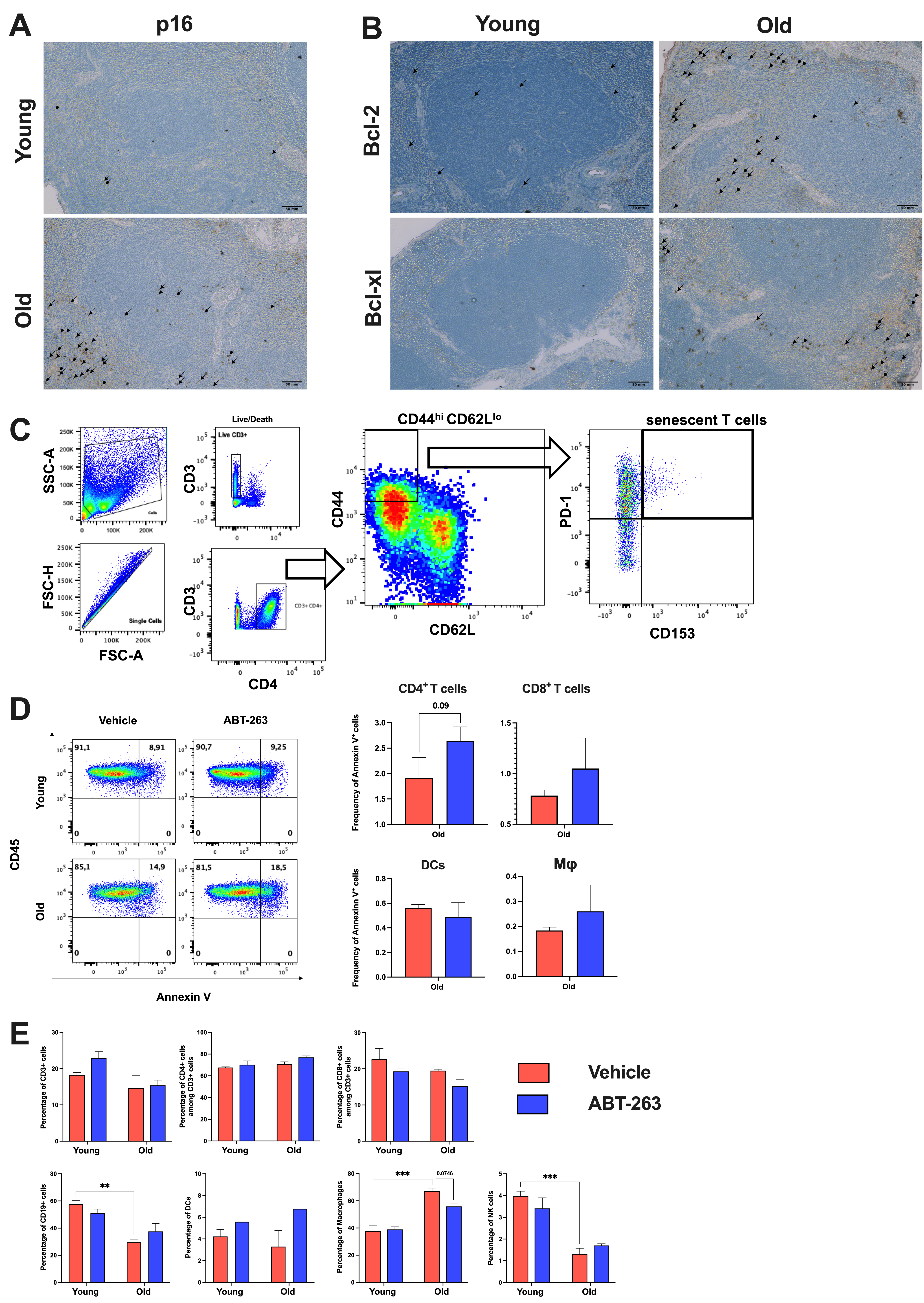

Supplement: Supplementary file 2 — Figure S2. [file ACEL-22-e14007-s001.tiff]

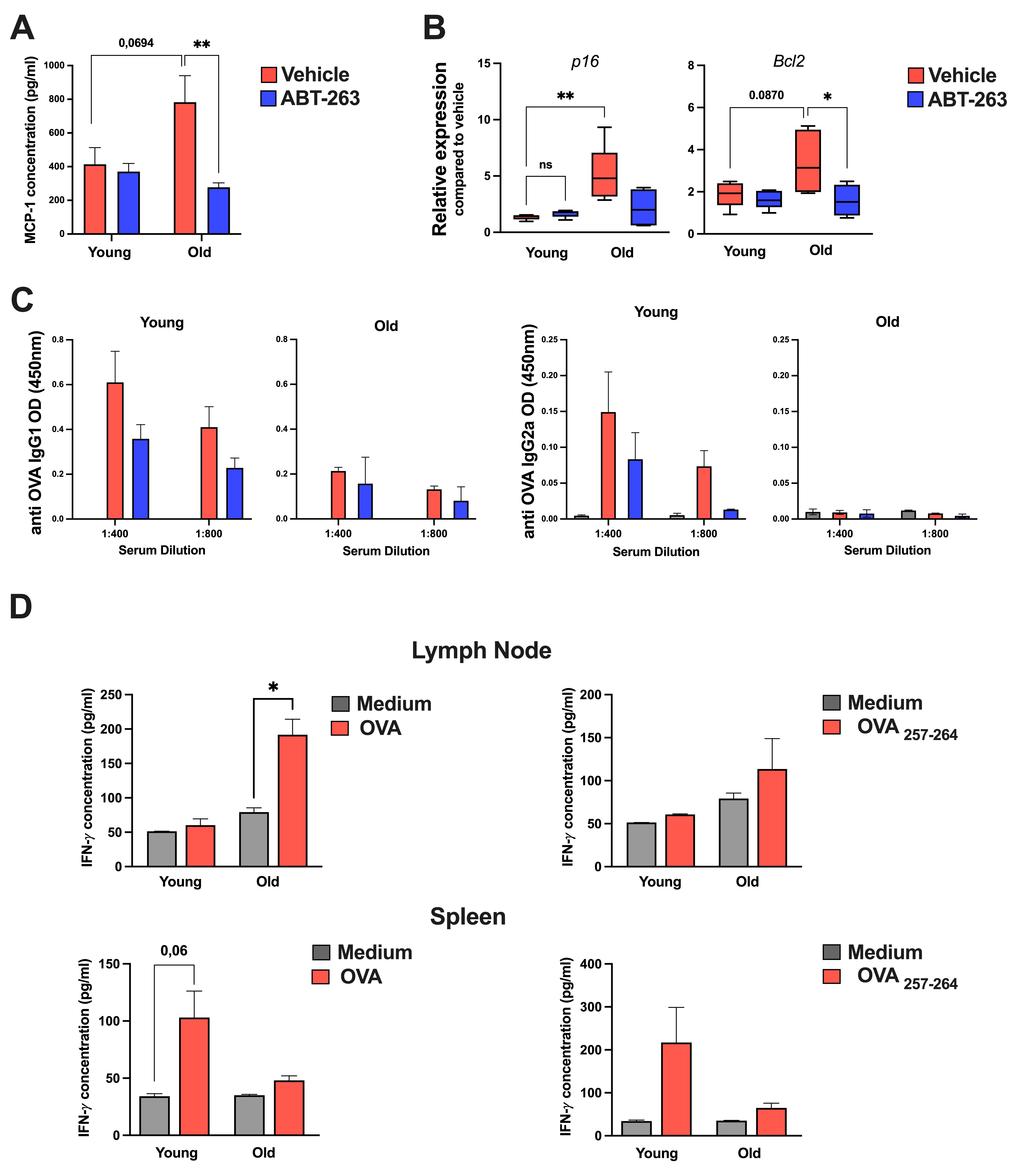

Supplement: Supplementary file 3 — Figure S3. [file ACEL-22-e14007-s002.tiff]

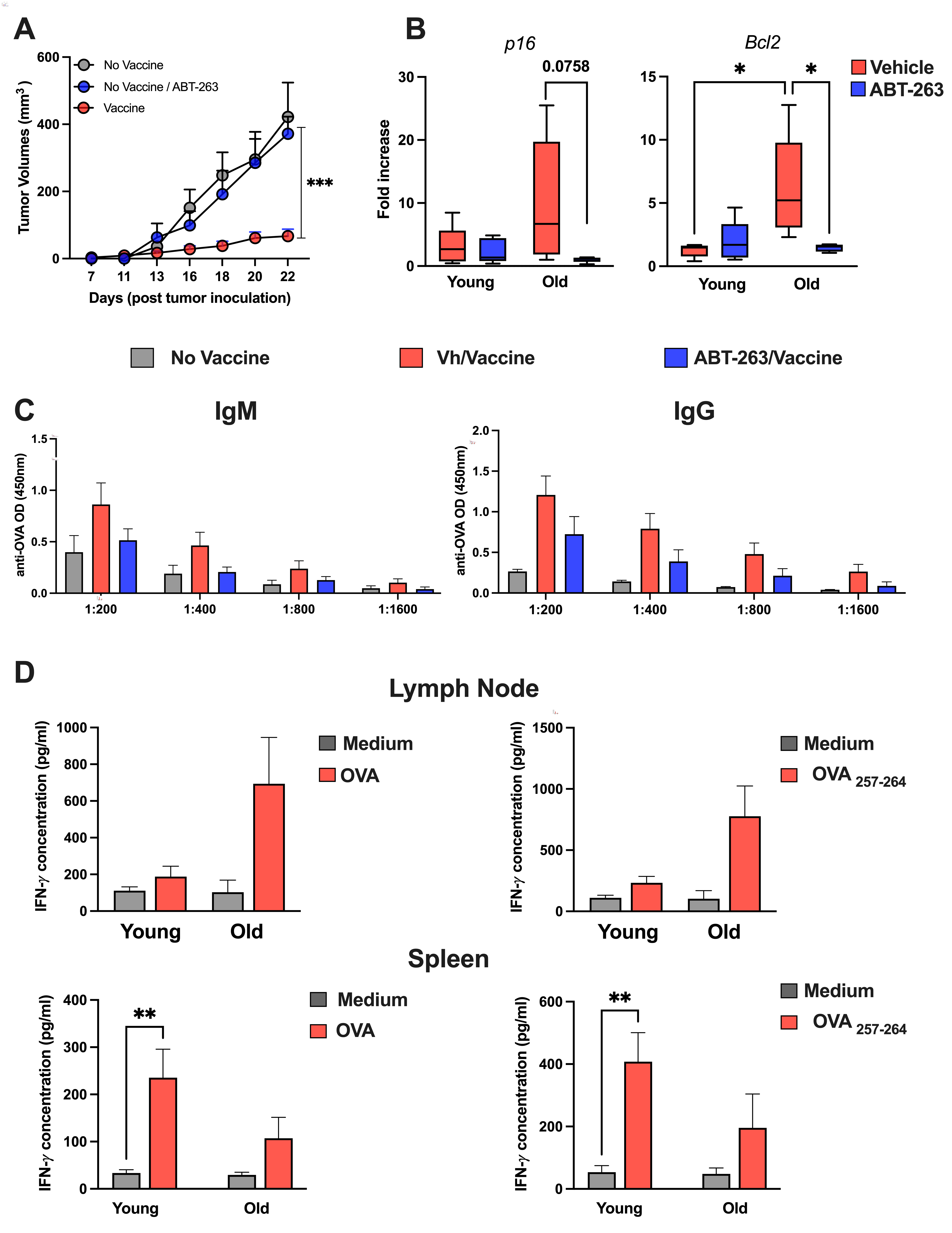

Supplement: Supplementary file 4 — Figure S4. [file ACEL-22-e14007-s004.tiff]
